# Effects of predictable behavioral patterns on anxiety dynamics

**DOI:** 10.1038/s41598-022-23885-4

**Published:** 2022-11-10

**Authors:** Martin Lang, Jan Krátký, Dimitris Xygalatas

**Affiliations:** 1 LEVYNA: Laboratory for the Experimental Research of Religion, Masaryk University, Brno, Czech Republic; 2 Department of Anthropology, University of Connecticut, Storrs, USA; 3 Department of Psychological Sciences, University of Connecticut, Storrs, USA

**Keywords:** Human behaviour, Stress and resilience

## Abstract

People face stressors that are beyond their control and that maladaptively perpetuate anxiety. In these contexts, rituals emerge as a natural coping strategy helping decrease excessive anxiety. However, mechanisms facilitating these purported effects have rarely been studied. We hypothesized that repetitive and rigid ritual sequences help the human cognitive-behavioral system to return to low-entropy states and assuage anxiety. This study reports a pre-registered test of this hypothesis using a Czech student sample (n = 268). Participants were exposed to an anxiety induction and then randomly assigned to perform one of three actions: ritualized, control, and neutral (no-activity). We assessed the effects of this manipulation on cognitive and physiological anxiety, finding that ritualized action positively affected anxiety decrease, but this decrease was only slightly larger than in the other two conditions. Nevertheless, the between-condition differences in the reduction of physiological anxiety were well-estimated in participants more susceptible to anxiety induction.

## Introduction

Anxiety is an adaptive response that facilitates precaution in uncertain environments and motivates people to avoid hazards^1^. Nevertheless, many environmental and social hazards are beyond human control, preventing individuals from taking necessary precautions and from extinguishing the anxiety system's unsettling activation^2^. Such pro-longed activation may have long-term negative health effects, increase the risk of suicide, or manifest in various psychological disorders^3–8^. Consequently, despite the lack of physical means to mitigate uncontrollable hazards, individuals are compelled to regain a sense of control over an uncertain future and decrease their anxiety^9^. To this end, they often adopt ritualized behavioral patterns aimed at resolving the threat, even though there is no clear functional relationship between the behavior and the potential threat^10^.

The use of ritual as a natural coping strategy in uncontrollable, anxiogenic situations (albeit not exclusively; see^11,12^) was already noted over a century ago by anthropologist Bronislaw Malinowski^13^. Since then, ample ethnographies, observational studies, and experiments demonstrated that ritualized behaviors tend to increase when people lack control over potentially threatening situations^14–18^. Malinowski^13^ further suggested that these naturally emerging behavioral patterns serve to assuage the anxiety triggered by prospective hazards. Scant experimental evidence supports this thesis^19–23^. For instance, in a naturalistic experiment, our past work showed that Hindu women had decreased anxiety after performing familiar ritual prayer^24^.

While these studies provide general support for the anxiolytic effects of ritual behavior, the cognitive mechanisms facilitating these effects remain understudied or confined to specific psychopathologies (such as Obsessive–Compulsive Disorder), hampering our understanding of how the human mind naturally deals with uncontrollable stressors. Moreover, cultural rituals vary widely in their structure, duration, and complexity^21^. This diversity makes it difficult to identify the specific cognitive and behavioral mechanisms underpinning those anxiolytic effects^11^. To introduce conceptual clarity and disentangle these culturally intricate behavioral patterns, several anthropologists suggested that apart from the culturally specific content of the ritual act, the ritualization of behavioral patterns itself may impact anxiety dynamics^2,14,25^.

Our previous work^24,26,27^ indicated that among the defining aspects of ritualized behavior, its redundancy, repetitiveness, and rigidity respond to anxiety and play crucial roles in its modulation. Specifically, redundancy refers to the fact that rituals are often superfluous from a pragmatic point of view (e.g., blessing a ship for safe sails). Repetitiveness and rigidity refer to the fact that rituals occur frequently in recurrent action sequences (e.g., repeating a prayer 50 times or circumambulating an altar seven times in a clockwise direction). As such, redundancy reflects the compulsion to engage in any behavioral act to avoid the uncontrollable threat, whereas repetitiveness and rigidity represent specific behavioral properties influencing the perceived control over the threat. We argued that they do so by regulating the internal entropy of the cognitive-behavioral system^27^.

In the entropy model of uncertainty^28^, frequent mismatches between expected and perceived sensory inputs caused by the vast spectrum of possible states of the world increase the internal entropy of the human cognitive-behavioral system^29^. Such increasing entropy threatens to destabilize the system. In anxiogenic situations where the occurrence and magnitude of the threat are uncertain or unknown^30,31^, higher levels of neuronal hierarchies in the human brain have difficulty generating precise predictions for lower neuronal hierarchies. The incoming sensory stimuli related to the potential threat do not match the expected representations at the lower neuronal levels, and prediction error propagates through the cortical hierarchy back to the higher neuronal levels^28^. To resolve the error, predictive success may be enhanced by active inference^32^, in which an individual searches for sensory stimuli that would either confirm the prior beliefs about future states of the world or help generate a new model for the incoming stimuli^33^. However, such active inference is difficult, if not impossible, in the case of uncontrollable threats (e.g., before an incoming environmental disaster).

We propose that the human cognitive-behavioral system deals with increasing entropy by returning to familiar, well-predictable states. Ritualized behaviors, defined by repetitive and rigid behavioral sequences, are a prime example of action leading to low-entropy states^27^. If anything, ritual is structure. By providing well-ordered predictable perceptual patterns, ritualized behavior helps minimize internal prediction error and decreases the internal entropy that is experienced as unnerving anxiety. Instead of actively sampling the state-space of threat-related stimuli, the sensory system is occupied by self-induced ritualized stimuli that perfectly match the predictions of the generative model in higher neuronal hierarchies. That is, in the case of ritualized behavior, the generative model samples predictions from a well-known probability distribution of habitual behaviors.

Testing this entropy-minimization model of ritualized behavior, we previously showed that inducing anxiety in a laboratory setting led to the increased occurrence of ritualized behavior, characterized by redundancy, repetitiveness, and rigidity of motor actions^27^, a finding wich was later conceptually replicated by Karl and Fischer^34^. Here, we report the results of a pre-registered experimental study that examined whether ritualized behavioral patterns help decrease anxiety. Modifying our original research design^27^, we investigated the link between ritualization and anxiety by exposing participants to an anxiogenic situation and then manipulating the type of activity they performed while anxious. Specifically, participants were exposed to one of three conditions: ritualized, control (non-ritualized activity), and neutral (no activity). We predicted that there would be a larger reduction in subjective as well as physiological anxiety from the pre-activity period to the post-activity period for those in the ritualized condition compared to those in the control and neutral conditions.

## Methods

### Participants

In a double-blind, between-subject design, we recruited 270 Czech and Slovak participants from Masaryk University's student pool. Based on our a priori power analysis, this sample size should allow us to detect a medium effect of our ritual manipulation with > 80% statistical power (see the pre-registration document). Two participants left the experiment before finishing, leaving us with 268 participants (M_age_ = 22.6; SD_age_ = 3.7; 162 women/104 men). Participants were randomly assigned to either a ritualized (n = 92), control (n = 85), or neutral (n = 91) condition. See SM, section S2.1, for exclusion criteria. The experiment was approved by the ethical committee of Masaryk University, and all procedures followed the relevant guidelines and regulations.

### Materials

**Figure 1:**
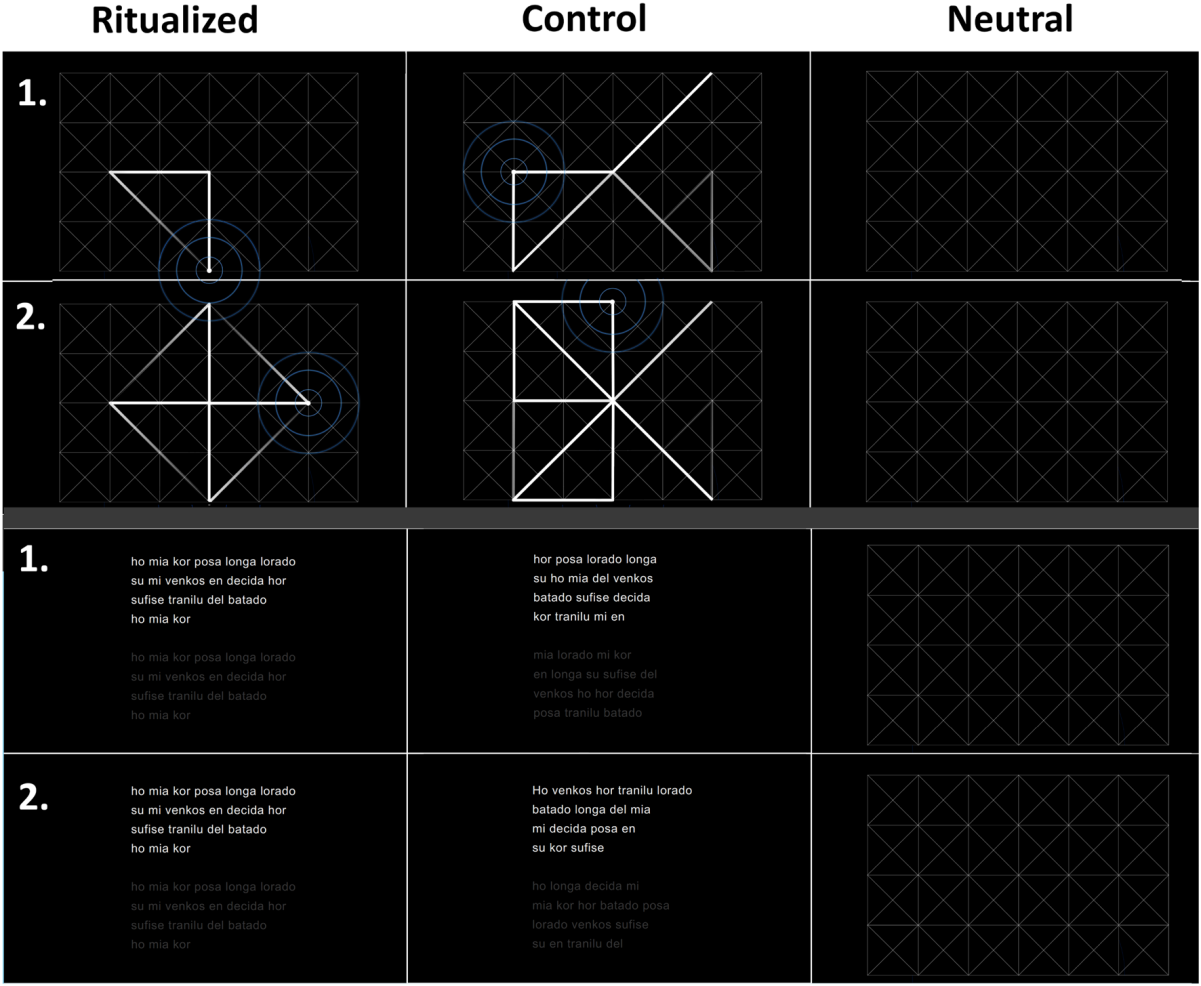
The motor and verbal tasks used to manipulate ritualization. Top two rows: motor task. In the ritualized condition, the dot moved predictably in rigid patterns, while in the control condition, the dot's movements were unpredictable, as illustrated by the trace line (which was not visible to participants). Steps 1 and 2 were sampled from the movement sequence to illustrate the dynamics of the moving dot. In the neutral condition, participants only watched the white grids without the moving dot. Bottom two rows: verbal task. In the ritualized condition, participants read aloud the same stanza for three minutes in the ritualized condition, while in the control condition, they read randomly ordered words from the same stanza. The second verse is grayed out so that participants know it will come (as in karaoke). In the neutral condition, participants again watched the same white grid as in the motor task.

To capture the key aspects of human ritual behavior, we used two sets of stimuli—motor and verbal. To avoid overwhelming participants by having them perform two types of unknown activities simultaneously, half of them performed a motor task, and the other half performed a verbal task. Both tasks manipulated repetitiveness and rigidity in the same way. In the case of the motor task, the experimental manipulation comprised two animated and one static video-clips presented on a screen (124 × 71 cm) positioned so that participants could comfortably reach the screen with their dominant hand while being seated. Each clip comprised a squared 12 × 8 white grid rendered on a black background. However, the ritualized and control conditions also included an animated dot that moved along the lines of the white grid. Participants in these conditions were asked to follow the dot with their dominant hand for three minutes. In the ritualized condition, the dot repeated a specific movement pattern in a rigid way, while in the control condition, the movements did not form any pattern. The number of movements was counterbalanced across these conditions. In the neutral condition, participants only watched the white grid without the moving dot for three minutes (see Fig. 1).

The verbal task was presented on the same screen with an identical neutral condition as in the motor task. In the ritualized and control conditions, participants were asked to read aloud a modified poem in Esperanto titled "Ho, mia kor" written by L. L. Zamenhof. We used the second stanza from this poem. Participants in the ritualized condition recited this stanza for three minutes. Those in the control condition recited stanzas in which the same words were randomly ordered for each stanza (see Fig. 1 for illustration and the OSF repository for all the used stimuli). We chose a poem in Esperanto to minimize the chance that participants would recognize the language and understand the meaning of specific words (only one participant out of the 91 who saw this manipulation recognized the language as Esperanto). This manipulation allowed us to focus solely on the structural aspects of the verbal act.

For both the motor and verbal tasks, we obtained two main dependent variables: self-reported anxiety as a subjective measure of cognitive anxiety, and electro-dermal activity (EDA) as a measure of physiological anxiety (obtained using a BIOPAC BioNomadix PPGED wireless module; Biopac Systems, Inc., Goleta, CA, USA). The subjective measure was assessed by asking participants, "Do you feel anxious right now?" on a visual analog scale ranging from 0 to 100 at intervals of one, anchored by "Definitely no" and "Definitely yes" at the ends of the scale. This measure was obtained three times during the experiment: (1) at baseline, (2) after the speech-preparation period, and (3) after the expectation period that followed the experimental manipulation (see “Procedure” for details). EDA was measured continuously throughout the experiment and the number of non-specific skin conductance responses (NS-SCRs) was calculated for each period of interest. The details on the processing of the EDA data are reported in SM, section S2.2.

In addition, we also asked participants how 'nervous' and 'stressed' they felt throught the experiment (same times as the subjective anxiety questions). These two words are frequently used in the Czech everyday language in anxiogenic contexts, so we used them to serve as comparisons assessing the robustness of the purported effects of ritualization on self-reported anxiety. Additional survey data included sex, age, experience with public speaking, trait anxiety (using the 'trait' version of the State-Trait Anxiety Inventory^35^; standardized Cronbach's alpha = 0.92), and the Desirability of Control scale^36^ (standardized Cronbach's alpha = 0.85). After the experiment, we asked participants whether they thought they would be presenting their speech on a scale from 0 to 100. All surveys were distributed on a laptop using the Survey Monkey app.

### Procedure

The experiment took place in a laboratory room at Masaryk University that comprised a chair, table, a screen, and a recording studio set up with a video camera and studio lights to increase participants' expectation that they would give a speech. Upon individually coming to the laboratory, participants read and signed an informed consent form, and a research assistant blind to our hypotheses placed the BioNomadix module on their non-dominant hands. Participants were instructed to remain seated with their non-dominant hand comfortably placed on their thighs. Next, they were asked to fill out the STAI and DoC questionnaires (including self-reported anxiety) and then sit in silence for three minutes, during which we obtained baseline physiological measurements (*baseline period*).

After the baseline measurement, a research assistant asked participants to prepare a five-minute speech about an art object placed in front of them (see the object used in^27^). They were told that this speech would be recorded on video and analyzed by a group of experts on public speaking. Moreover, in the verbal task, they were told that there was a chance they would not be required to give the speech (this change was implemented to avoid unnecessary deception as required by the managers of the participant database we sampled from—we account for this change analytically in our models, see below). Participants were informed that they had three minutes to prepare the speech. After the anxiety-inducing *speech-preparation period*, participants reported on their current anxiety, nervosity, and stress. This speech-preparation period served as the reference category for cognitive and physiological anxiety in our statistical models.

Next, participants were assigned to one of the three conditions, determined by randomization conducted before the onset of data collection. Participants in all conditions were seated in front of the screen. In the ritualized and control conditions of the motor task, they were asked to follow a moving dot on the screen with their dominant hand. In the same conditions in the verbal task, they were asked to read aloud a text as it appeared on the screen. A short demonstration and training preceded the main task. Those in the neutral conditions were asked to watch the screen. Participants were given no reason for this activity, which lasted three minutes (*manipulation period*).

After the task, participants were asked to remain seated for three minutes, waiting to present their speech. Three minutes later, they were asked to report on their current anxiety, nervosity, and stress. This *expectation period* served to measure the change in cognitive and physiological anxiety from the speech-preparation period. Following the expectation period, participants in the motor version of the experiment were informed that a random-number-generator did not select them to give the speech. Participants in the verbal version rolled a die. If the outcome was between 1 and 5, the research assistant informed participants that they were not selected to speak in front of the video camera. If the die roll yielded 6, participants performed their speech, which was recorded and rated but not further analyzed. Participants did not know about their probability of being selected to give the speech.

**Figure 2:**
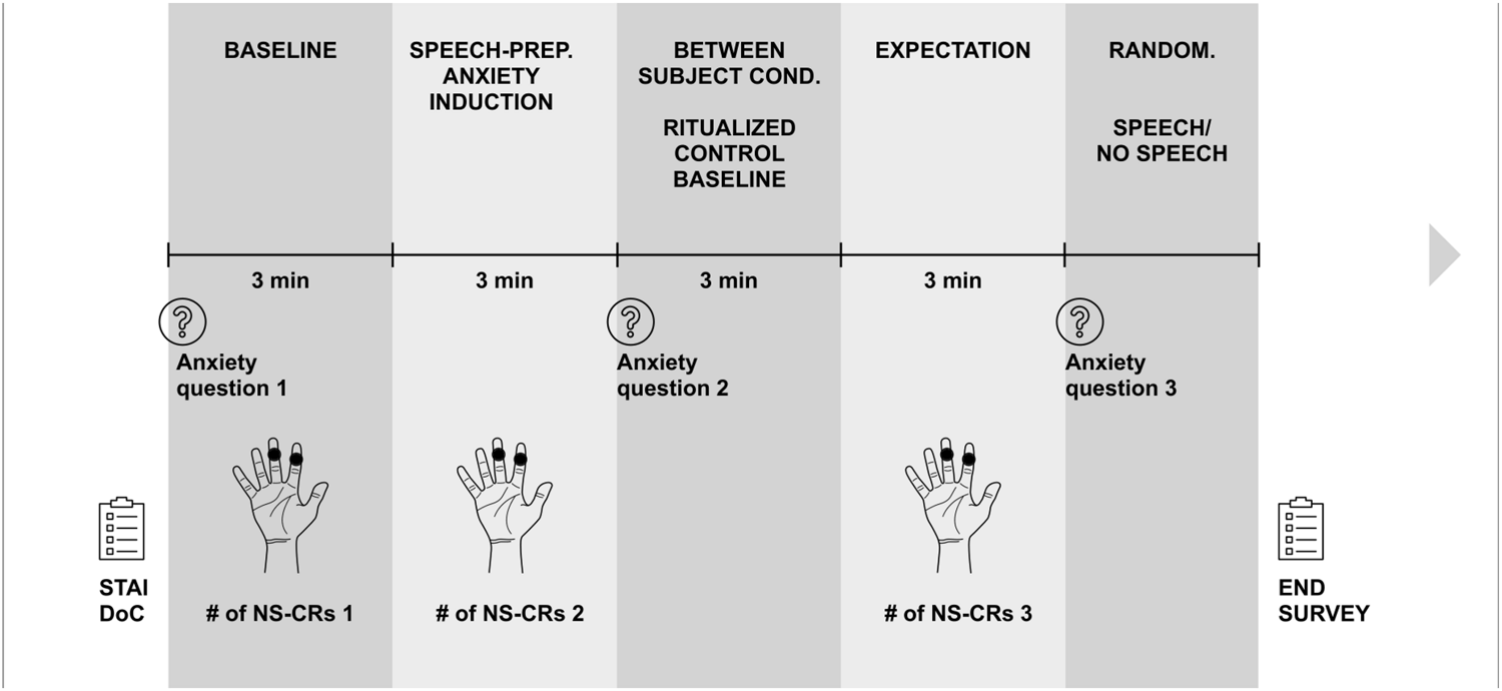
Procedure overview. Our main analyses compared anxiety decrease between the speech-preparation (measure 2) and expectation (measure 3) periods while holding constant the individual baseline anxiety levels (measure 1). NS-SCRs = Non-Specific Skin Conductance Responses; STAI = State-Trait Anxiety Inventory; DoC = Desirability of Control.

Finally, participants filled out a demographic survey and were asked questions related to the experimental stimuli. At the conclusion of the study, they received 150 CZK as compensation for their participation. For a visual overview of the design, see Fig. 2.

## Results

### Pre-registered analyses

Data were analyzed in R, version 4.0.1^37^, using generalized linear mixed models (GLMMs) with the Tweedie distribution for the self-reported data and negative binomial distribution for the NS-SCRs data (see SM, section S2.3 for details on model selection and distributional assumptions and Table S1 for raw means and standard deviations of cognitive and physiological anxiety measures).

#### Anxiety induction check

As expected, our induction of anxiety was captured by both the cognitive and physiological measures. On a scale of 0 to 100 for self-reported anxiety, the GLMM predicted an increase in self-reported anxiety from 12.2 (baseline period) to 23.7 points (speech-preparation period), an effect that was well estimated (β = 0.66, 95% CI = [0.51, 0.81]). Despite this increase, the statistical mode of self-reported anxiety in the speech-preparation period was 0, suggesting that our anxiety induction did not affect cognitive anxiety for all participants. There were no substantial between-condition differences in the increase of cognitive anxiety from the baseline to the speech-preparation period (β_ritual vs. control_ = 0.02, 95% CI = [− 0.36, 0.40]; β_ritual vs. neutral_ = 0.04, 95% CI = [− 0.32, 0.41]).

We also observed a well-estimated increase from the baseline to the speech-preparation period in our measure of physiological anxiety—the number of NS-SCRs (β = 0.40, 95% CI = [0.36, 0.45]). This increase was relatively higher in the ritualized condition compared to the control condition, although the 95% CI of this difference contained zero (β_ritual vs. control_ = − 0.10, 95% CI = [− 0.21, 0.01]; β_ritual vs. neutral_ = − 0.04, 95% CI = [− 0.15, 0.07]). The model estimated on average 23 NS-SCRs during the three-minute baseline period and 34.4 NS-SCRs during the speech-preparation period.

#### Effects of ritual on cognitive anxiety

**Figure 3:**
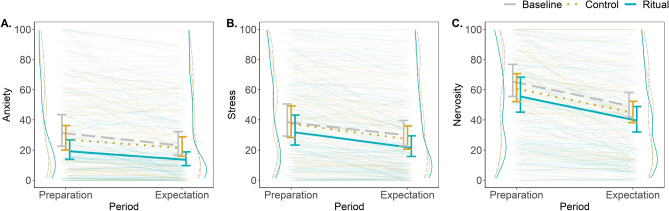
Estimated between-condition differences for the self-reported measure of anxiety (**A**), stress (**B**), and nervosity (**C**) on the full sample. Each plot includes density curves representing the raw distribution of data per each period and condition; raw data from all participants illustrating the dynamics of anxiety, stress, and nervosity; and estimated slopes with 95% CI for the between-condition differences. Data include the motor and verbal tasks.

In terms of our main pre-registered analyses, we observed a well-estimated decrease in cognitive anxiety from the speech-preparation to the expectation period in the ritualized condition (β = − 0.34, 95% CI = [− 0.49, − 0.19]), averaging to 5.5 points on the self-reported anxiety scale. As predicted, this decrease was lower in the control and neutral conditions compared to the ritualized condition (i.e., less negative; β_ritual vs. control_ = 0.11, 95% CI = [− 0.09, 0.31]; β_ritual vs. neutral_ = 0.06, 95% CI = [− 0.13, 0.24]). However, the wide confidence intervals of these estimates do not allow us to draw confident inferences beyond the study sample. Adding theoretically important variables to the model did not change the observed between-condition differences in anxiety dynamics (see Fig. 3A for an illustration of the detected effect and Table 1 for the estimates from the full model).

**Table 1:** Beta-Estimates from GLMM with 95% CI (the pre-registered analyses).

	Self-reported anxiety	NS-SCRs
Condition: control	0.136 (− 0.227, 0.499)	− 0.019 (− 0.110, 0.073)
Condition: neutral	0.241 (− 0.114, 0.596)	− 0.090 (− 0.180, 0.001)
Task: verbal	− 0.128 (− 0.415, 0.159)	− 0.048 (− 0.119, 0.023)
Sex: woman	0.009 (− 0.300, 0.319)	− 0.039 (− 0.112, 0.035)
STAI (0–80)	0.059 (0.041, 0.077)	0.001 (− 0.003, 0.005)
DoC (0–100)	0.014 (− 0.004, 0.031)	0.006 (0.001, 0.010)
Experience presenting (0–100)	− 0.010 (− 0.016, − 0.005)	− 0.001 (− 0.002, 0)
Period: expectation	− 0.343 (− 0.493, − 0.193)	− 0.404 (− 0.462, − 0.346)
Control*period	0.11 (− 0.091, 0.311)	0.032 (− 0.051, 0.114)
Neutral*period	0.056 (− 0.137, 0.248)	0.059 (− 0.023, 0.141)
Constant	2.978 (2.599, 3.356)	3.618 (3.529, 3.708)
*µ*_*intercept*_ participant	1.799	0.055
*µ*_*slope*_ participant	0.001	< 0.001
N participants	245	237
N observations	490	474

Self-reported anxiety was modeled using the Tweedie distribution with log link while the number of NS-SCRs was modeled using the negative binomial distribution with log link. Condition shows the difference in the value of the outcome variable in the speech-preparation period between the ritualized condition and the control and neutral conditions, respectively. Task shows the difference between the motor and verbal tasks. STAI = State-trait anxiety inventory. DoC = Desirability of Control scale. STAI, DoC, and Experience were centered at their means. Period shows the difference in the outcome variables between the speech-preparation and expectation periods in the ritualized condition. Control*Period shows the difference in the slopes of the period variable between the ritualized and control conditions and Neutral*Period shows the difference in the slopes of the period variable between the ritualized and neutral conditions (our main comparison). *µ*_*inttercept*_ Participant is the variance explained by varying intercepts by participants. *µ*_*slope*_ Participant is the variance explained by varying the slopes of baseline anxiety by participants.

#### Effects of ritual on physiological anxiety

**Figure 4:**
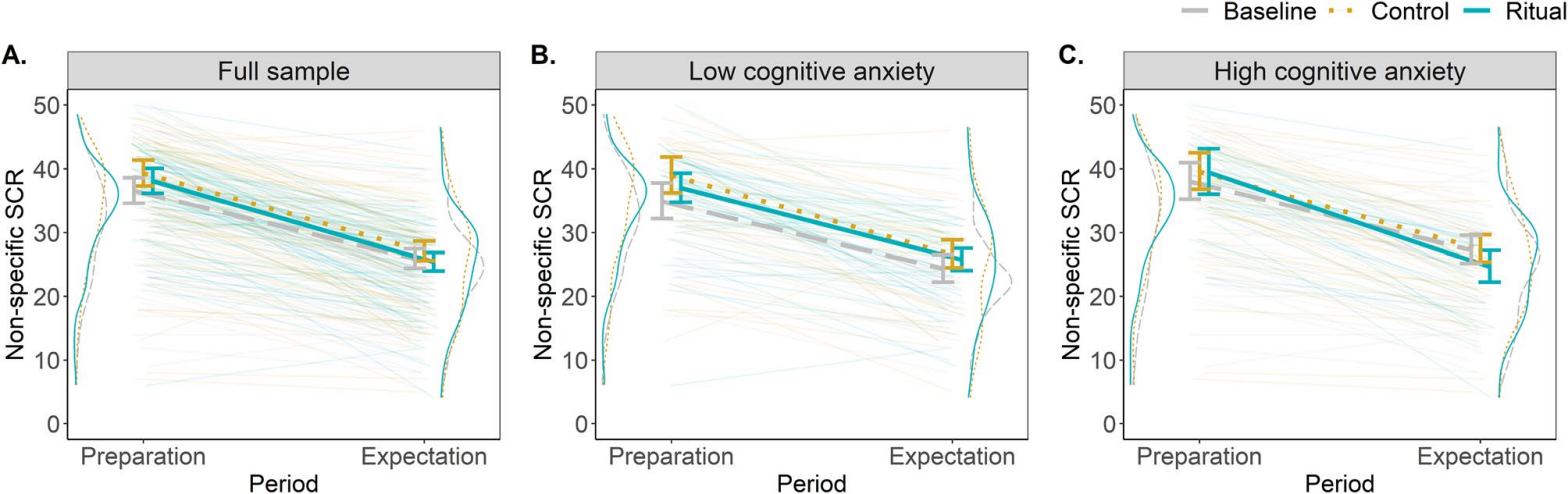
Estimated between-condition differences for the physiological measure of anxiety on the full sample (**A**), on the half of the sample with lower cognitive anxiety (**B**), and on the half of the sample with higher cognitive anxiety (**C**). Each plot includes density curves representing the raw distribution of data per each period and condition; raw data from all participants illustrating the dynamics of physiological anxiety; and estimated slopes with 95% CI for the between-condition differences. Data include the motor and verbal tasks.

Similarly to the results of cognitive anxiety, the decrease in the number of NS-SCRs from the speech-preparation to the expectation period was estimated to be 12.7 in the ritualized condition (β = − 0.41, 95% CI = [− 0.46, − 0.35]), and this decrease was lower in the control and neutral conditions (i.e., less negative; β_ritual vs. control_ = 0.03, 95% CI = [− 0.05, 0.12]; β_ritual vs. neutral_ = 0.06, 95% CI = [− 0.02, 0.14]). While most of the probability mass was above zero for the comparison of the ritualized and neutral conditions, the 95% CIs still contained zero, indicating an imprecisely estimated effect. This remained true after adding the theoretically important controls to the model (see Fig. 4A for an illustration of the detected effect and Tab. 1 for the estimates from the full model).

### Exploratory analyses

#### Effects of ritual on stress and nervousness

Apart from self-reported anxiety, we also asked participants how stressed and nervous they felt throughout the experiment. Stress decreased from the speech-preparation period to the expectation period in the ritualized condition (β = − 0.39, 95% CI = [− 0.52, − 0.27]), and this decrease was again smaller in the other two conditions, with the neutral condition having more precisely estimated differences (β_ritual vs. control_ = 0.07, 95% CI = [− 0.10, 0.25]; β_ritual vs. neutral_ = 0.16, 95% CI = [− 0.01, 0.33]). On the other hand, while compatible with previous results, the between-condition differences in the measure of nervosity revealed poorly estimated effects (β_ritual vs. control_ = 0.03, 95% CI = [− 0.12, 0.18]; β_ritual vs. neutral_ = 0.06, 95% CI = [− 0.08, 0.21]). See Fig. 3B,C.

#### Effects of rituals on concurrent cognitive and physiological anxiety

We explored whether the effects of ritualized behavior on physiological anxiety would be greater in people experiencing cognitive anxiety. This analysis was motivated by a finding from our previous study^27^, where increasing physiological anxiety predicted dispersion in our measure of repetitive motor patterns. That is, while physiological anxiety was associated with higher behavioral repetitiveness, this was not true for all anxious participants, and we speculated that despite increased physiological anxiety, some participants did not display repetitive motor patterns because their cognitive appraisal of anxiety was not sufficiently destabilizing. In line with this proposition, we examined whether the between-condition differences in physiological anxiety decrease would be greater in people who experienced more cognitive anxiety during speech preparation.

To explore this hypothesis, we divided our sample into two counterbalanced halves based on the cognitive anxiety experienced during speech preparation (n = 120/118; dividing cognitive anxiety score = 24). As expected, there was no between-condition difference in the decrease of physiological anxiety for participants in the low cognitive anxiety group (β_ritual vs. control_ = − 0.02, 95% CI = [− 0.13, 0.09]; β_ritual vs. neutral_ = 0.00, 95% CI = [− 0.11, 0.12]). However, we observed the predicted effect in the high cognitive anxiety group (β_ritual vs. control_ = 0.11, 95% CI = [− 0.02, 0.23]; β_ritual vs. neutral_ = 0.14, 95% CI = [0.02, 0.26]). See Fig. 4B,C. While supportive of our prediction, this result is only exploratory and based on a halved sample size; further evidence is needed to support the important role of cognitive anxiety in the effects of ritualized behavior on physiological anxiety.

## Discussion

We hypothesized that distinctive structural aspects of rituals, namely their repetitiveness and rigidity, may be responsible for the previously documented effects of ritual behavior on anxiety alleviation in uncertain situations. The results of our pre-registered analyses revealed that while the decrease in both cognitive and physiological anxiety was larger in the ritualized compared to the control and neutral conditions, these differences were poorly estimated and did not allow for inference beyond the study sample. Comparable results were obtained for the additional perceived stress and nervosity measures, with the former showing a larger difference between the ritualized and neutral conditions. However, after splitting the sample into halves based on the reported cognitive anxiety during speech preparation, we obtained well-estimated between-condition differences in physiological anxiety.

Despite the between-condition differences generally being in the predicted direction, the latter result suggests that the relationship between ritualization and anxiety is more nuanced and mostly confined to states that are characterized by cognitive anxiety. In conjunction with the results of our previous study^27^, we suggest that physiological anxiety leads to higher ritualization only when people simultaneously experience high cognitive anxiety. As a result, only in these situations would people experience the beneficial effects of ritualized behavior on alleviating physiological anxiety. Since a sizable portion of our sample did not perceive the anxiety induction as anxiogenic (or, at least, did not report it as such), the overall effect of ritualization on anxiety might have been masked and detected only in people affected by our anxiogenic task.

This interpretation may be further supported by the underlying predictive processing framework. While the prediction error generated at the lower hierarchy of neuronal structures increases physiological anxiety via the sympathetic axis of the autonomic nervous system (manifested as increased NS-SCRs in our case), the activation of the sympathetic axis does not necessarily enter perceptual consciousness^38^. If individuals have strong priors immunizing them to the effects of a specific threat (e.g., experience with public speaking) or are low on trait anxiety, the interoceptive inputs signaling stress may be evaluated as noisy or imprecise and given reduced attention^39^. In the absence of cognitive anxiety, people may not be compelled to take protective action, that is, to ritualize. When asked to ritualize, the lack of need for protective action might forestall prediction-error minimization at the lower neuronal level hierarchies that generate physiological anxiety. In contrast, individuals for whom public speaking is stressful weigh interoceptive signals of threat more heavily, resulting in heightened cognitive anxiety^40^. The amplified prediction error from lower neuronal hierarchies would be then susceptible to exteroceptive signals generated by predictable ritualized action, effectively decreasing physiological anxiety. This conclusion has important implications, as it suggests that ritualization is beneficial precisely in those situations where anxiety-reduction strategies are needed.

A complementary explanation of the current findings might suggest that while rituals help decrease anxiety related to uncertain threats, the prediction-error minimization facilitated by ritualized action is only one mechanism in a synergetic bundle of other mechanisms present in cultural rituals and, as such plays, only a limited role^24^. For instance, the belief that rituals are effective in mitigating the anxiety-provoking threat may further boost the effects of ritualized behavior. Indeed, research shows that repetitive and rigid behaviors are perceived as more effective^41,42^ and simply telling participants that they are performing an efficient ritual facilitates anxiety decrease even if the movement sequence is identical^43^. Moreover, in our study, we used an impromptu performed repetitive behavior unknown to participants, which is in stark contrast to how rituals are usually performed, that is, as sequences of movements and verbalizations learned through long-term practice, often from a very young age. When performed weekly or even several times a day, rituals become familiar embodied acts that may act as strong priors and have a larger effect on the minimization of prediction error. Indeed, this interpretation is consistent with the fact that we found only weak effects in the current study but stronger effects when studying real-life ritual practices^24^.

The conclusions from the current study are limited in several ways. First, as displayed in Figs. 3 and 4, our method for inducing anxiety had variable effects on participants. While it should be expected that anxiety sensitivity is variable in the population, this variability potentially hampers inference on anxiety alleviation mechanisms since some participants did not experience sufficient anxiety during speech preparation. We dealt with this issue by dividing our sample into low and high anxious groups, but such a step necessarily decreases statistical power. Increasing the sample size to accommodate the non-responsive participants and using more complex diagnostic criteria (e.g., intolerance of uncertainty) should provide more nuanced insights into the personality types that could potentially benefit from ritualization. Furthermore, rather than sampling from the general population, we recruited university students, who may have substantial public speaking experience and be peculiar in other ways. Finally, the detected differences between conditions were rather small and poorly estimated, questioning whether our manipulation of predictable action was salient enough.

Two modifications of our design may introduce a stronger test of the presented hypothesis. First, having participants practice the ritualized action at home and memorize it could amplify the effects of ritual rigidity. If the performed action is not novel but a learned response to an anxiogenic situation (as most rituals in anxiogenic contexts are), this enhanced rigidity should be more effective in minimizing prediction error^44^. Second, increasing the task duration might have also produced larger effects. While the duration of the verbal and motor tasks was informed by our previous study^27^, the effects of ritualization on anxiety may strengthen with time until reaching a plateau, similar to other ritual effects^45^. Moreover, the most efficient duration of ritualization likely varies between individuals, as does the length of individual ritual performance in the real world^24^.

These limitations should be best resolved by a combination of laboratory and field studies, where laboratory experiments aim to manipulate specific de-contextualized mechanisms (e.g., varying the length of ritual performance)^27,34^ and field studies investigate the phenomenon in its natural context^24^. Such complementary evidence would help us better understand naturally occurring anxiolytic behaviors that are readily available and may help regain higher levels of functioning and promote mental health and well-being in the face of adversity. This would be especially important in populations that are at risk of chronic anxiety but do not have access to biomedical or other professional interventions.

## Supplementary Information

Supplementary Information.

## Data Availability

Data and R code are publicly available at the OSF repository: https://osf.io/p8bas/.
