# Supplementary material for: Effects of predictable behavioral patterns on anxiety dynamics
Source: Sci Rep. 2022 Nov 10;12:19240. doi: 10.1038/s41598-022-23885-4 (PMC9649661; doi:10.1038/s41598-022-23885-4)
Supplement: Supplementary file 1 — Supplementary Information. [file 41598_2022_23885_MOESM1_ESM.pdf]

Effects of predictable behavioral patterns on anxiety dynamics

Martin Lang[1*], Jan Krátký[1], & Dimitris Xygalatas[2,3]

[1] LEVYNA: Laboratory for the Experimental Research of Religion, Masaryk University, Czech Republic

[2] Department of Anthropology, University of Connecticut, USA

[3] Department of Psychological Sciences, University of Connecticut, USA

*Corresponding author: Martin Lang; martinlang@mail.muni.cz

Supplementary Material

S1. Supplementary tables

Table S1. Means and standard deviations of self-reported anxiety and non-specific skin conductance responses across conditions and periods.

Condition	Period	Anxiety M (SD)	NS-SCR M (SD)
Ritual	Baseline	14.07 (19.38)	23.61 (9.24)
	Preparation	25.57 (26.66)	36.40 (7.76)
	Expectation	18.42 (21.38)	24.28 (7.09)
Control	Baseline	17.67 (21.06)	25.78 (11.22)
	Preparation	32.65 (28.60)	36.00 (9.88)
	Expectation	26.10 (24.93)	24.82 (8.63)
Neutral	Baseline	21.05 (23.81)	22.76 (10.36)
	Preparation	40.18 (32.90)	33.55 (9.10)
	Expectation	30.56 (28.62)	23.77 (7.78)

S2. Supplementary information

S2.1. Exclusion criteria

After data collection, 14 participants were removed from the analyses: two due to the suspicion that they knew about the procedure from previous participants; seven due to procedural mistakes (e.g., shorter periods of interest, questionnaires distributed at wrong times) and five due to unexpected disturbances (e.g., a rock concert taking place outside the lab). Furthermore, four participants were removed from the analysis of the self-reported anxiety data due to technical problems (e.g., issues with the internet connection), and 12 participants were removed from the analysis of the EDA data due to technical problems during recording (e.g., uncalibrated electrodes or excessive hand movement despite explicit instructions to remain still). The number of participants used to test each hypothesis is specified in our regression models.

S2.2. EDA processing

Physiological anxiety was indexed by the number of non-specific skin conductance responses (NS-SCRs; Boucsein, 2012; Boucsein et al., 2012). The EDA data were obtained using a BIOPAC BioNomadix PPGED wireless module (Biopac Systems, Inc., Goleta, CA, USA) connected to a BIOPAC MP160 system, which records data through the Acq*Knowledge* software (version 5.0.4) installed on a Windows PC. The wireless module was placed on the wrist of participants' non-dominant hand, with three cables attached to the distal phalanges of the index, middle, and ring fingers. The index and middle fingers were used to measure EDA using disposable electrodes and EDA gel to increase the EDA signal's strength. The third cable recorded Photo-Pletysmo-Graphy (PPG) using a reusable transducer. However, while we aimed to use the PPG data in the analysis, those data were heavily skewed by the respiratory rate (including the measure of pulse-rate variability), and there was a substantial decrease in the quality of the PPG signal after our anxiety induction (presumably due to anxiety-related vasoconstriction limiting blood flow to participants' fingers). Hence, we do not include these data in

the current study and recommend that researchers rely on the standard measure of heart-rate variability in studies inducing anxiety. EDA data were recorded at a 2000 Hz frequency, then de-sampled to 62.5 Hz to facilitate analysis and cleaned by applying a low-pass filter fixed at 1 Hz. The phasic signal was constructed using the smoothing baseline removal function of the Acq*Knowledge* software with a 5-sec window. Changes in the phasic signal crossing the 0.01 µS threshold were identified as NS-SCRs. The number of NS-SCRs was obtained during the three-minute baseline period, three-minute speech-preparation period, and three-minute expectation period. We discarded the first and last 5 seconds from each period, which often included artifacts due to transitions between these different recording periods.

S2.3. Analysis details

We first examined the likely data-generating process for the two outcome measures. As expected (see our pre-registration document), the number of NS-SCRs revealed overdispersion, and the negative binomial distribution fitted better than the Poisson distribution. Unexpectedly, self-reported anxiety did not follow a normal distribution, with the highest density around 0 and a long tail to 100. Thus, we used the Tweedie distribution (Dunn & Smyth, 2005), which estimates a 'p' parameter to fit the mixture of Poisson and Gamma distributions, which resemble the data-generating process bound at 0 with a long tail. This distribution fitted well to our data. All models were fit using the glmmTMB package (M. E. Brooks et al., 2017), their performance checked using the DHARMa package (Hartig, 2019), estimates converted using the effects package (Fox, 2003), and plotted using the ggplot2 package (Wickham, 2016).

Given our mixed design (PERIOD [baseline/speech preparation/expectation] x CONDITION [neutral, control, ritual]), we first assessed the effects of our anxiety induction, comparing the increase in cognitive and physiological anxiety from the baseline period to the speech preparation period. We tested whether there was an increase in anxiety across all conditions and then compared the anxiety slopes between conditions, assuming no substantial differences. As our primary analysis, we compared the differences in anxiety dynamics from the speech-preparation period to the expectation period between the ritualized and control conditions and between the ritualized and neutral conditions. In this generalized linear mixed model (GLMM), we interacted the experimental period (speech preparation vs. expectation) with condition (ritualized vs. control; ritualized vs. neutral), holding constant the effects of the modality of our manipulation (motor vs. verbal), varying intercepts by participants to account for the repeated nature of our measures, and letting the slopes of baseline anxiety to vary by participants to account for the differential effects of baseline anxiety on anxiety induction (this is not mentioned in the pre-registration since we did not expect baseline anxiety to differ between conditions). Finally, to test the stability of the detected effects, we added to this model further predictors that have theoretical importance for anxiety dynamics, including participants' sex, the STAI and DoC measures, and experience with public speaking (see the R code at the OSF repository for exact model specifications).

S2.4. Additional measures

Participants in the ritualized and control conditions were asked whether they perceived their movements as some kind of ritual in the motor task or whether the verbal task felt like reciting a prayer. We also asked participants in the motor task how random they perceived the movements to be, but this question raised puzzlement among several participants. Thus, rather than asking about the randomness of stanzas' composition, we asked about the repetitiveness of verbal stimuli after the verbal task. Finally, participants were prompted to share what they thought the study was about.
